# Bridging Text and Speech for Emotion Understanding: An Explainable Multimodal Transformer Fusion Framework with Unified Audio–Text Attribution

**DOI:** 10.3390/jintelligence13120159

**Published:** 2025-12-03

**Authors:** Ashutosh Pandey, Jasmeet Singh, Maninder Kaur

**Affiliations:** Computer Science Engineering Department, Thapar Institute of Engineering and Technology, Patiala 147001, Punjab, India; apandey_me23@thapar.edu (A.P.); jasmeet.singh@thapar.edu (J.S.)

**Keywords:** emotion, multimodal learning, explainable AI, speech, text fusion

## Abstract

Conversational interactions, rich in both linguistic and vocal cues, provide a natural context for studying these processes. In this work, we propose an explainable multimodal transformer framework that integrates textual semantics (via RoBERTa) and acoustic prosody (via WavLM) to advance emotion understanding. By projecting both modalities into a shared latent space, our model captures the complementary contributions of language and speech to affective communication, achieving an 0.83 accuracy value across five emotion categories. Crucially, we embed explainable AI (XAI) techniques including Integrated Gradients and Occlusion to attribute predictions to specific linguistic tokens and prosodic patterns, thereby aligning computational mechanisms with human cognitive processes of emotion perception. Beyond performance gains, this work demonstrates how multimodal AI systems can support transparent, human-centered emotion recognition.

## 1. Introduction

Human social interactions rely heavily on socioemotional skills, such as the recognition, expression, and regulation of emotions, which are central components of social cognition. Social cognition refers to the mental processes that enable individuals to perceive, interpret, and respond to social information, including others’ emotions, intentions, and behaviors. These abilities are essential for building empathy, maintaining relationships, supporting learning, and promoting mental health across the lifespan. A lack of well-developed socioemotional perception has been linked to difficulties in communication, reduced academic and professional performance, and vulnerabilities to psychological distress. With the rise of artificial intelligence, new opportunities have emerged to study and enhance socioemotional understanding through computational models that integrate linguistic and paralinguistic cues, offering novel insights into how emotions are communicated and perceived. This perspective aligns with research in neuroscience and psychology emphasizing the centrality of social cognition in human interactions, while recent AI studies have begun incorporating insights from human emotion perception to improve recognition models Liu et al. (2023).

Human emotion is intrinsically multimodal, conveyed not only through linguistic content but also through an array of non-verbal cues such as prosody, intonation, rhythm, pitch, and pauses. In natural communication, these verbal and vocal elements operate in tandem, shaping how affective states are perceived and interpreted. Text alone captures the semantic dimension of communication, yet omits the paralinguistic signals that often carry substantial emotional significance. For instance, the phrase “I’m fine” may convey reassurance when spoken with a calm tone, sarcasm when delivered with a flat tone, or frustration when expressed with a sharp and elevated pitch—distinctions that are inaccessible without acoustic information.This is consistent with evidence from neuroscience showing that prosody is a crucial channel for emotional decoding Lehnen et al. (2025).

Multimodal emotion recognition seeks to address this limitation by integrating complementary information from multiple channels, most notably text and speech. Transformer-based language models such as RoBERTa have demonstrated exceptional capabilities in capturing semantic nuances, discourse-level context, and syntactic dependencies within textual data. In parallel, speech encoders such as WavLM or HuBERT have proven effective in extracting prosodic and paralinguistic features from audio signals, capturing variations in pitch, energy, and spectral dynamics that are closely tied to emotional expression.

Advances in large-scale pretrained models have made it feasible to obtain rich, high-level embeddings from both modalities and to combine them within a unified framework. Fusion strategies, whether performed at the feature level, decision level, or within a shared latent space, enable the integration of complementary verbal and vocal cues, leading to more robust and contextually aware emotion understanding. Empirical evidence consistently shows that multimodal architectures outperform unimodal counterparts, particularly in spontaneous and contextually rich conversational settings where emotional signals are distributed across both linguistic and acoustic domains.

The development of such systems holds significant promise for applications in healthcare, education, social robotics, and customer service, where accurate and context-sensitive emotion recognition can enhance user interaction and trust. Importantly, coupling multimodal architectures with interpretability frameworks ensures that these models remain transparent and accountable, enabling practitioners to trace predictions back to the linguistic and acoustic features that informed them.

In this work, we propose a transformer-based framework for emotion understanding using multiple data modalities that integrates RoBERTa-derived textual embeddings with WavLM-derived acoustic embeddings. The modality-specific representations are projected into a shared latent space and fused to jointly model semantic and paralinguistic cues. Experimental results demonstrate that the proposed approach achieves significant enhancement in both accuracy and macro F1-score across five emotion categories, highlighting the value of combining text and audio for robust and interpretable emotion understanding.

The core contributions of this article are summarized as follows:

1. Development of transformer-based emotion understanding framework using multimodal (text + audio) inputs.
2. Application and adaptation of XAI techniques—including token attribution to dissect and visualize the internal mechanisms of fine-tuned transformer models.
3. Comparative analysis of modality contributions, highlighting the importance of prosodic cues in speech-based emotion understanding and their interplay with textual features.
4. Empirical and interpretive insights into how affective information is encoded and decoded across different modalities and transformer layers, contributing to the growing body of work in transparent AI.

## 2. Literature Survey

Early speech emotion recognition (SER) systems relied on hand-crafted acoustic features such as MFCCs, pitch, energy, and formants, which were input to conventional classifiers like Gaussian mixture models or support vector machines. Tits et al. (2018) advanced this paradigm by repurposing a deep automatic speech recognition (ASR) model as a feature extractor for emotion prediction. Their approach leveraged high-level acoustic representations from the ASR network to predict emotional dimensions such as valence and arousal, outperforming traditional handcrafted features like eGeMAPS. Notably, they found that features from earlier ASR layers, closer to raw audio, were more informative for arousal, while later layers, aligned with textual content, correlated with valence. This study demonstrated the value of transfer learning in SER, bridging handcrafted and learned features, and offering interpretable insights into how different layers capture emotion-relevant cues in speech. With the rise of machine learning and deep learning, speech emotion recognition (SER) has increasingly shifted toward end-to-end models that automatically learn emotion-relevant features from raw audio data. Various models like recurrent neural networks (RNNs),convolution neural networks (CNNs) and transformers applied to speech signals, with a major research focus on improving neural architectures to better capture the nuances of emotional expression. For instance, Locumber and Fabian (2020) evaluated traditional and machine learning methods for emotion classification by first applying feature engineering to extract relevant features and then training a deep neural network using 40 MFCC coefficients. Kaloub and Abed Elgabar (2024) explored emotion classification using audio-based machine learning, applying classifiers such as K-Nearest Neighbours, Random Forest, Sequential Minimal Optimization (and Simple Logistic, while emphasizing the benefits of combining acoustic features like MFCC and Mel spectrograms to improve detection accuracy. Siva et al. (2024) focused on machine learning techniques for emotion classification in audio data using LSTM networks, decision trees, and CNNs, incorporating data augmentation to increase model robustness and generalizability. Arjun et al. (2024) discussed the role of traditional ML methods such as logistic regression and SVM, alongside an examination of critical spectral and acoustic features vital for effective audio-based emotion recognition. Similarly, Pai and Punnath (2024) adopted both traditional machine learning models—including multilayer perceptrons, decision trees, SVMs CNNs and LSTM, showing that deep models significantly enhanced raw audio feature extraction and classification accuracy. Jain et al. (2024) demonstrated the superiority of a 2D CNN model resembling VGG19 for audio emotion classification over traditional approaches, utilizing log-Mel spectrograms with data augmentation instead of conventional MFCC features. Furthermore, Zou et al. (2022) proposed an end-to-end SER model that leverages a co-attention mechanism to fuse multi-level acoustic features, effectively emphasizing complementary aspects of speech and achieving notable gains in recognition accuracy. Eriş and Akbal (2024) advanced Speech Emotion Recognition (SER) by proposing a hybrid approach that fuses deep learned features from a transformer-based model (wav2vec 2.0) with hand-crafted features. Their method employs iterative feature selection on each set, followed by a majority-voting ensemble, yielding a high accuracy on a multi-corpus dataset with a notable improvement over deep features alone. This demonstrates the complementary value of traditional features and offers interpretability into crucial acoustic characteristics. Chowdhury et al. (2025) developed a lightweight ensemble of deep models for Speech Emotion Recognition (SER), utilizing established acoustic features like MFCCs and chroma as input to compact CNN and CNN-BiLSTM networks. This approach achieved competitive accuracy with significantly fewer parameters than larger deep models, demonstrating that high SER performance can be attained with reduced computational cost through judicious feature selection and efficient network design. The reliance on interpretable, human-understandable features also provides a degree of model interpretability. Both Eriş and Akbal (2024) and Chowdhury et al. (2025) underscore an evolution in SER methods where pure end-to-end learning is not the only option—instead, combining traditional feature knowledge with deep learning can yield both accuracy and some transparency.

As Speech Emotion Recognition (SER) models grow in complexity, researchers are increasingly integrating explainable AI (XAI) methods to enhance interpretability and trustworthiness. A notable pioneering effort by Nfissi et al. (2023) introduced an explainability-driven feature boosting approach for SER, which iteratively refines acoustic feature sets using Shapley values to identify and prune less important features. This not only boosts accuracy, as validated on the TESS dataset where it surpassed human-level recognition, but also provides clear insights into feature contributions, highlighting XAI’s pivotal role in guiding SER model development. Extending this work, Nfissi et al. (2024)’s “Unveiling Hidden Factors” applied this iterative Shapley-value-based feature boosting across multiple SER benchmarks, consistently improving accuracy and outperforming state-of-the-art deep learning methods. This integration of XAI enhanced performance by eliminating spurious features and offered valuable interpretability, confirming the importance of known emotionally relevant acoustic features and increasing model transparency. In contrast to Nfissi et al.’s approach of infusing explainability into model creation, Kim and Kwak (2024) explored post hoc explainability for deep-learning-based SER. They fine-tuned pretrained deep networks for high-accuracy emotion classification and then applied Grad-CAM, LIME, and occlusion sensitivity to interpret these models. This allowed for the generation of heatmaps and perturbation-based explanations on audio inputs, visualizing the model’s focus (e.g., higher frequencies for “angry” speech, slower pitch for “sad”)—findings that align with human acoustic knowledge, build trust, and identify areas for improvement. Their work underscores the crucial role of post hoc XAI for robust and deployable SER systems. Collectively, these studies Kim and Kwak (2024) and Nfissi et al. (2023, 2024) signify a crucial evolution in SER research, moving beyond solely improving recognition rates to a greater emphasis on understanding model behavior and ensuring intuitively valid decisions. By integrating explainable AI, researchers are better equipped to trust SER systems and pinpoint the speech characteristics truly indicative of human emotions, paving the way for SER technologies that are both effective and transparent.

Recent advancements in Speech Emotion Recognition (SER) emphasize multimodal fusion and hybrid approaches, moving beyond unimodal analysis. Kim et al. (2023) demonstrated the superiority of a hybrid multimodal system, leveraging complementary information from diverse sources like audio and visual cues to significantly outperform single-modality models. This trend is further contextualized by Zhang et al. (2024) systematic review of multimodal deep-learning-based emotion identification, which highlights the efficacy of deeply-learned features and model-level fusion, particularly via cross-modal attention, while also pinpointing persistent challenges such as computational complexity, the “black-box” nature of deep models, and the need for enhanced fusion strategies and solutions for data scarcity. Building on this, Zhang et al. (2023) introduced MER-HAN, a novel framework employing hybrid attention networks for audio and text inputs that utilizes distinct encoders, a Cross-Modal Attention block, and a Multimodal Emotion Classification block to achieve superior performance on IEMOCAP and MELD, though acknowledging its current limitation to only audio and text. Similarly, Singh et al. (2021) proposed a hierarchical deep learning approach for SER, integrating hand-crafted audio features with contextualized text embeddings to achieve strong performance across unimodal and multimodal scenarios, despite limitations in architectural generalization and broader modality integration. These works, alongside Eriş and Akbal (2024) demonstration of enhanced robustness and accuracy through fusing deep and handcrafted features, underscore a significant shift towards hybrid models balancing performance with transparency. Further extending the capabilities of multimodal fusion, Li et al. (2022) introduced a context-aware framework using WavLM-Large and BERT, refining features with Squeeze-and-Excitation and a convolutional context module before alignment via a Multimodal Transformer, yielding strong performance on IEMOCAP. Hosseini et al. (2024) presented a trimodal MER model integrating CNN-LSTM audio features, Inception-ResNet-v2 facial expressions, and Bi-LSTM Word2vec text embeddings, using sequential binary fusions and Chi-square feature selection to achieve notable accuracy on IEMOCAP, though facing limitations in generalizability and handling contextual dependencies. Chen et al. (2022) tackled noise and modality imbalance with a Key-Sparse Transformer combining RoBERTa and wav2vec 2.0 embeddings via stacked Cross-Modal Attention Blocks, achieving competitive results on IEMOCAP. Lastly, Wang et al. (2025) enhanced both accuracy and interpretability through a label-guided transformer model that fuses semantic and tonal label embeddings via cross-attention, with ablation studies confirming the benefits of emotion-aligned attention and interpretability constraints, further solidifying the importance of multimodal and interpretable approaches in modern SER. Table 1 provides the comparative key literature contributions in speech emotion recognition.

## 3. Methods

**Figure 1:**
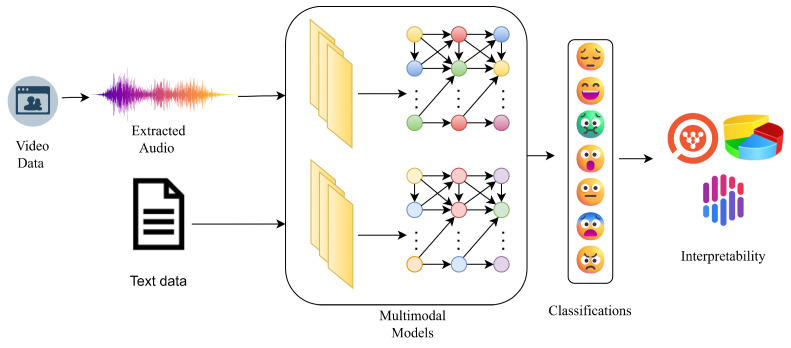
System overview for multimodal approach.

The proposed system aims to perform multimodal emotion understanding by leveraging both audio and textual information extracted from video data. As illustrated in Figure 1, the pipeline begins with raw video inputs, from which audio signals are extracted and transcribed text is obtained. These modalities are processed independently through modality-specific transformer-based models, which encode the audio and text inputs into high-dimensional representations. For the audio modality, we extract waveform features from the video stream and process them using pretrained speech models such as WavLM or Wav2Vec2.

Concurrently, the textual transcripts associated with the dialogues are tokenized and passed through a pretrained language model such as RoBERTa. These unimodal embeddings are then projected into a common latent space through dimensionality reduction techniques to ensure compatibility across modalities.The aligned embeddings from both modalities are fused within a multimodal neural network architecture that learns joint representations. This fusion makes the model to acquire cross-modal linkages and improves the robustness of emotion prediction. The final output layer produces categorical emotion classifications, typically covering five discrete emotional states such as joy, rage, astonishment, sorrow, and indifference To enhance transparency and trust in model predictions, the system incorporates interpretability modules. XAI techniques such as Integrated Gradients for text and Occlusion Sensitivity for audio are applied to attribute emotion predictions to specific input features, allowing for both quantitative and qualitative analysis of the model’s decision-making process.

### 3.1. Dataset Specifications

The work utilizes a Multimodal Emotion Lines Dataset (MELD) that serves as a robust resource for the study of multimodal emotion recognition, leveraging spontaneous dialogue from the television show “Friends.” The dataset provides two primary modalities: textual transcripts of utterances and corresponding video clips. Each of the dataset’s 12,462 utterances is meticulously tagged with seven distinct emotion categories anger, disgust, fear, joy, sadness, surprise, neutral and indifference as well as sentiment labels (positive, negative, or neutral). Although the source dataset originally contained seven emotion labels only five were used in the final experiments, as the disgust and fear categories contained critically low samples. A crucial feature of MELD is the inclusion of conversational context, which enables analysis of how emotions evolve throughout a dialogue. The corpus is systematically divided into a training set of 10,000 utterances, a validation set of 1109, and a test set of 1353, facilitates standardized evaluation and comparison of models.

### 3.2. Audio Extraction and Enhancement

To create the audio corpus, soundtracks from *N* (9990) video recordings were extracted and converted to single-channel WAV files at a sampling rate of 48 kHz. All resampling was performed with an anti-aliasing low-pass filter, a standard practice implemented in the torchaudio library, to prevent spectral distortion and aliasing artifacts during the rate conversion process.Given the importance of high-quality audio for accurate emotion understanding, all extracted waveforms were subsequently processed using DeepFilterNet v2 Schröter et al. (2022). This real-time neural speech enhancement model was employed with its default pretrained weights and configurations, including an FFT size of 512, a hop size of 128, and a Hann window with a 5 ms look-ahead. The model enhanced each audio file by applying learned spectral masking filters, which effectively suppressed background noise while preserving essential prosodic and spectral cues critical for emotion classification. Following this enhancement step, the waveforms were downsampled to 16 kHz to align with the input requirements of the WavLM feature extractor.

### 3.3. Dataset Preparation

The denoised audio files were aligned with the original text transcripts, affective state labels. The resulting corpus constituted a fully synchronized, bimodal dataset (audio and text) for each utterance. The CSV was first loaded into and any rows missing critical fields such as *Utterance*, *Emotion*, *Dialogue ID*, or *Utterance ID* were discarded. A unique filename for each utterance was constructed by concatenating its dialogue and utterance identifiers, and non-alphanumeric characters in the textual utterance were removed to normalize white space and punctuation.

### 3.4. Data Preprocessing

To ensure label reliability, the emotion categories Disgust and Fear were excluded from the dataset due to extremely limited sample availability, making it infeasible to train a stable classifier without severe class imbalance. Moreover, these classes exhibited low inter-annotator agreement indicating reduced label reliability. Therefore to preserve the dataset integrity and classification robustness, both categories were omitted from the final experiment. For each remaining utterance, the corresponding WAV file was located by concatenating the root audio directory with its generated filename. Entries with missing audio files were systematically discarded to maintain dataset consistency and integrity. During the preprocessing stage, the enhanced waveforms were loaded using the torchaudio library. Each signal was converted to mono and resampled from its original 48 kHz rate to 16 kHz using a sinc-based interpolation algorithm with anti-aliasing filters. This step was crucial to match the input requirements of the downstream feature extractor. Subsequently, each signal underwent mean–variance normalization to standardize the data Algorithm 1.
**Algorithm 1** Audio extraction and enhancement pipeline1:**procedure** EnhanceAudio($V_{\mathrm{dir}},A_{\mathrm{dir}}$)2:    $F\leftarrow \mathrm{ListFiles}(V_{\mathrm{dir}})$3:    N←Length(F)4:    /* Extract audio from each video */5:    **for** i=1 **to** *N* **do**6:         f←F[i]7:         **if** *f* ends with “.mp4” **then**8:             $v_{\mathrm{path}}\leftarrow V_{\mathrm{dir}}+f$9:             $a_{\mathrm{path}}\leftarrow A_{\mathrm{dir}}+\mathrm{RemoveExt}\left(f\right)+.\mathrm{wav}$10:             RunFFmpeg($v_{\mathrm{path}},a_{\mathrm{path}}$)11:         **end if**12:    **end for**13:    /* Initialize DeepFilterNet */14:    CloneRepo(DeepFilterNetRepo)^1^15:    InstallDependencies(requirements.txt)16:    (M,S,−,−)←InitDF(())17:    /* Upload and preprocess audio */18:    $x_{\mathrm{noisy}}\leftarrow$UploadAudio (())19:    $\left(a,f_{s}\right)\leftarrow$LoadAudio(*x*_noisy_)20:    **if** $f_{s}\neq 48,000$ **then**21:         a←Resample$\left(\left(\right)a,f_{s},48,000\right)$22:         $f_{s}\leftarrow 48,000$23:    **end if**24:    /* Enhance audio */25:    $a_{\mathrm{tensor}}\leftarrow$ToTensor(()a)26:    $a_{\mathrm{tensor}}\leftarrow \mathrm{AddBatchDim}\left(a_{\mathrm{tensor}}\right)$27:    $a_{\mathrm{enhanced}}\leftarrow$Enhance$\left(\left(\right)M,S,a_{\mathrm{tensor}},f_{s}\right)$28:    $a_{\mathrm{enhanced}}\leftarrow \mathrm{RemoveBatchDim}\left(a_{\mathrm{enhanced}}\right)$29:    /* Save output */30:    WriteAudio(“enhanced_audio.wav”, $a_{\mathrm{enhanced}},f_{s}$)31:    **return** “enhanced_audio.wav”32:**end procedure**

## 4. Tokenization and Dataset Construction

Feature extraction was a critical step in converting raw audio and text data into informative numerical representations for subsequent modeling. For the audio modality, denoised utterances were processed directly as raw waveform samples using the WavLM-Base-Plus model. Each audio signal was resampled to 16 kHz, with mean variance normalization per-utterance applied to standardize the data. The waveforms were then zero-padded or truncated to a fixed length of 8 seconds (128,000 samples) before being fed into the model. The WavLM model subsequently transformed these normalized waveforms into 768-dimensional contextualized speech embeddings.

In parallel, textual utterances underwent preprocessing to remove non-alphanumeric characters. These cleaned texts were then tokenized using RobertaTokenizerFast (roberta-base). The tokenizer maintained case, utilized byte-level BPE subword segmentation, and generated token sequences that were truncated or padded to a fixed length of 64 tokens. These token sequences were then mapped to 768-dimensional embeddings via the pretrained RoBERTa encoder. The resulting standardized and context-aware representations from both modalities provided a robust foundation for accurate affective state recognition.

### 4.1. Model Architectures

**Figure 2:**
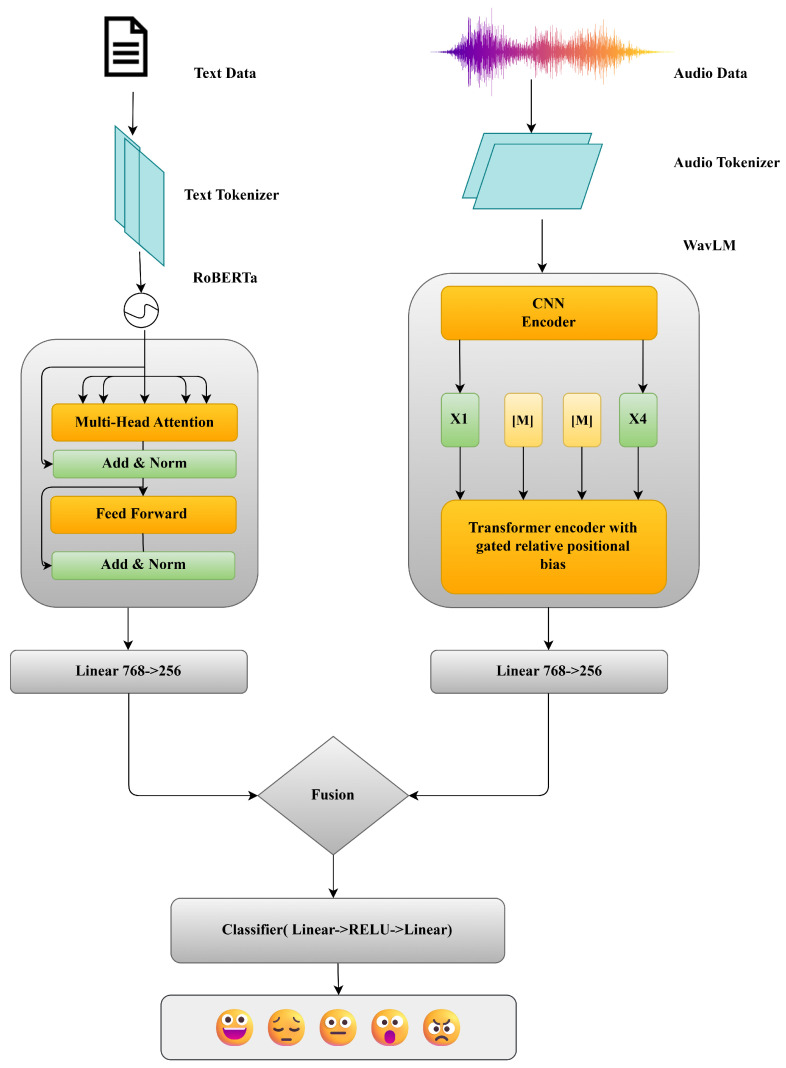
Multimodal architecture.

To harness large-scale self-supervised representations, the proposed system integrates two transformer-based backbones: WavLM for audio encoding and RoBERTa for text encoding Figure 2. Although both follow the canonical *“embeddings → stacked transformer”* paradigm, In this paradigm, raw inputs are first mapped into dense vector representations using subword lookup embeddings for text and linear or convolutional feature projections for speech, followed by positional encodings to preserve sequence order Algorithm 2. The resulting embedding sequence is processed by a deep stack of Transformer layers, where multi-head self-attention captures long-range contextual interactions and position-wise feed-forward networks iteratively refine representations, supported by residual connections and normalization for stable optimization. This architecture enables hierarchical contextual modeling and has become the de facto backbone for scalable self-supervised learning across both language and speech. A detailed, layer-wise exposition of the two models follows.
**Algorithm 2** Preprocessing, training, and evaluation for multimodal affective state classification1:**procedure** Multimodal Emotion Classification2:    D←ReadCSV(CSV_PATH)3:    D←DropNA(D,[Utterance,Emotion,Dialogue_ID,Utterance_ID])4:    D.file←Concat(“dia”,D.Dialogue_ID,“ttu”,D.Utterance_ID)5:    D.Utterance←Apply(clean_text,D.Utterance)6:    D←FilterOut(D,D.Emotion∈{disgust,fear})7:    D.filepath←Map(f↦JoinPath (AUDIO_DIR,f+“.wav”),D.file)8:    D←FilterExists(D,filepath)9:    (labels,le)←LabelEncode(D.Emotion)10:    wav_proc←Wav2Vec2Processor(“facebook/wav2vec2-base-960h”)11:    tok←RobertaTokenizerFast(“roberta-base”)12:    D←CreateDataset(D,wav_proc,tok)13:    $\left(D_{\mathrm{train}},D_{\mathrm{val}}\right)\leftarrow \mathrm{RandomSplit}\left(D,\;\left[0.8,0.2\right]\right)$14:    $L_{\mathrm{train}}\leftarrow$DataLoader$\left(D_{\mathrm{train}},\;batch\_size=8,\;shuffle=True,\;collate\_fn\right)$15:    $L_{\mathrm{val}}\leftarrow$DataLoader$\left(D_{\mathrm{val}},\;batch\_size=8,\;shuffle=False,\;collate\_fn\right)$16:    M←MultimodalEmotionClassifier(|le.classes|)17:    ℓ←CrossEntropyLoss()18:    $opt\leftarrow \mathrm{AdamW}(M.\mathrm{parameters}\left(\right),\;lr=2\times 10^{-5})$19:    $\left(M,\;opt,\;L_{\mathrm{train}},\;L_{\mathrm{val}}\right)\leftarrow \mathrm{accelerator}.\mathrm{prepare}\left(M,opt,L_{\mathrm{train}},L_{\mathrm{val}}\right)$20:    **For** e←1 to *E*                          ▹ — Training phase —21:    **For** batch $\in L_{\mathrm{train}}$22:    opt.zero_grad()23:    out←M(batch.inputs)24:    loss←ℓ(out,batch.labels)25:    accelerator.backward(loss)26:    opt.step()27:    **EndFor**                                 ▹ — Validation phase —28:    total_val_loss,val_preds,val_labs←0,[],[]29:    **For** batch $\in L_{\mathrm{val}}$30:    out←M(batch.inputs)31:    loss←ℓ(out,batch.labels)32:    total_val_loss+=loss33:    Append predictions and labels34:    **EndFor**35:    Compute metrics and print losses/accuracies36:    **If** total_val_loss<optimal_val_loss37:    optimal_val_loss←val_loss,wait←038:    **Else**39:    wait+=140:    **EndIf**41:    **EndFor**                              ▹ — Final evaluation & plotting —42:    PlotConfusionMatrix(val_labs, val_preds, le.classes)43:    PrintClassificationReport(val_labs, val_preds, le.classes)44:    PlotCurves(train_loss, val_loss, train_accuracy, val_accuracy)45:    **return** M,{train_loss,total_val_loss,train_accuracy,val_accuracy}46:**end procedure**

#### 4.1.1. WavLM-Base

WavLM, an extension of the Wav2Vec2.0 framework, is employed as the audio encoder in the proposed system. It enhances representation learning through the integration of masked prediction and denoising pretraining, thereby capturing both speaker-specific characteristics and semantic content with improved fidelity. This study utilizes the “base+” variant of WavLM, which follows a modular architecture. Input audio signals, sampled at 16 kHz, are first standardized and segmented into fixed-length frames via a convolutional feature extractor. This front-end comprises seven sequential one-dimensional convolutional layers with kernel sizes of [10, 3, 3, 3, 3, 2, 2] and strides of [5, 2, 2, 2, 2, 2, 2], effectively downsampling the input while preserving salient acoustic information.

The resulting frame-level representations are processed by a stack of Transformer encoder layers. Within each layer, a multi-head self-attention mechanism enables each time step to attend to all others, capturing both local and global temporal dependencies. Each 768-dimensional frame is linearly projected into query, key, and value vectors, and processed across twelve attention heads operating on 64-dimensional subspaces. Scaled dot-product attention is computed and normalized via a softmax function, with dropout (probability 0.1) applied to mitigate overfitting. The contextually weighted value vectors are then concatenated and re-projected to the original dimensionality. Following self-attention, each frame is forwarded into a position-wise feed-forward network (FFN), comprising of two linear transformations with an intermediate GELU activation. The FFN expands the input dimension from 768 to 3072 and subsequently projects it back to 768, with dropout (0.1) applied after the final projection for additional regularization. To ensure stable and efficient training across the deep encoder stack, WavLM adopts a pre-layer normalization and residual connection scheme. Each sublayer—self-attention and FFN—is preceded by layer normalization and wrapped in a residual connection, which facilitates gradient flow and preserves identity mappings. These architectural components collectively enable WavLM to learn robust and expressive acoustic representations, well-suited for downstream affective state classification tasks.

#### 4.1.2. RoBERTa

For the textual modality, we employ RoBERTa, a pretrained transformer-based architecture optimized for context-rich natural language understanding. Each input utterance is first tokenized into subword units using a byte-level Byte-Pair Encoding (BPE) tokenizer, which maintains a vocabulary of approximately 50,000 tokens. This encoding strategy balances vocabulary size and coverage by allowing frequent tokens to be represented as whole units while decomposing rare words into interpretable subunits. Each resulting token ID is mapped to a 768-dimensional vector through a learned embedding matrix, capturing distributional semantics and syntactic patterns learned from large-scale pretraining corpora. Since transformers are inherently order-agnostic, positional information is incorporated via learned 768-dimensional positional embeddings. These are added element-wise to token embeddings to encode both relative and absolute position within the sequence, allowing the model to distinguish between identical tokens in different contexts. The combined embeddings are then passed through a stack of twelve identical Transformer encoder layers, each composed of two sublayers: multi-head self-attention and a position-wise feed-forward network (FFN). In the self-attention mechanism, token representations are linearly projected into queries, keys, and values, and processed in twelve parallel attention heads, each operating over a 64-dimensional subspace. Scaled dot-product attention is computed for each head, followed by softmax normalization and dropout (0.1), and the resulting weighted value vectors are concatenated and projected back to 768 dimensions. This allows each token to integrate contextual information from all others in the sequence. The FFN then processes each token embedding independently through a two-layer feed-forward network: the input is expanded from 768 to 3072 dimensions, activated via a Gaussian Error Linear Unit (GELU), and projected back to 768 dimensions. Dropout with a probability of 0.1 is applied to the output to mitigate overfitting. To ensure training stability and facilitate gradient propagation, both sublayers employ residual connections and post-layer normalization. Specifically, the output of each sublayer is added to its input and then normalized to zero mean and unit variance. This design allows the network to iteratively refine intermediate representations while preserving useful input features. Ultimately, the final hidden state of the “[CLS]” token from the twelfth layer serves as a fixed-length, 768-dimensional embedding that summarizes the entire utterance, forming the basis for downstream emotion classification.

### 4.2. Dimensionality Reduction

To reconcile the heterogeneous representations produced by the audio and text encoders, each 768-dimensional embedding is linearly projected into a common 256-dimensional subspace. Formally, let $a\in R^{768}$ denote the audio embedding derived from WavLM, and $t\in R^{768}$ the text embedding derived from RoBERTa. The projections are defined as:

(1) $$a^{\prime }=W_{a}a+b_{a},\;t^{\prime }=W_{t}t+b_{t},$$

where $W_{a},W_{t}\in R^{256\times 768}$ and $b_{a},b_{t}\in R^{256}$ are learned parameters. This transformation ensures that both modalities are represented in a unified subspace, facilitating modality alignment and reducing computational complexity in subsequent layers.

### 4.3. Feature Fusion

The projected audio and text features, $a^{\prime }$ and $t^{\prime }$, are concatenated to form a 512-dimensional multimodal vector:

(2) $$m=\left[a^{\prime };\;t^{\prime }\right]\in R^{512}.$$

To improve generalization and prevent co-adaptation of features, a dropout layer with a probability p=0.3 is applied to the fused embedding *m*.

### 4.4. System Output

The regularized multimodal embedding *m* is fed into a two-layer feed-forward neural network producing class logits. The first layer applies a ReLU activation:

(3) $$h_{1}=\mathrm{ReLU}\left(W_{1}m+b_{1}\right),\;W_{1}\in R^{256\times 512},\;b_{1}\in R^{256}.$$

The second layer projects the hidden representation to the affective state classification space of dimension *C*:

(4) $$\hat{y}=W_{2}h_{1}+b_{2},\;W_{2}\in R^{C\times 256},\;b_{2}\in R^{C}.$$

The output logits $\hat{y}$ are fed into a softmax activation to get class likelihood. This late fusion architecture effectively captures and integrates complementary emotional cues from both modalities, enabling robust multimodal affective state classification.

### 4.5. Explainability Techniques

For elucidating the computational framework’s predictions and assess feature relevance within the audio modality, we employ two post hoc explainability methods: **Integrated Gradients (IG)** and **Occlusion** Algorithm 3.

*Integrated Gradients* Sundararajan et al. (2017) is a gradient-based attribution method that quantifies the importance of each input feature by integrating the gradients from a baseline input $x^{\prime }$ (typically zero) to the actual input x. The attribution for feature $x_{i}$ is computed as:

(5) $${\mathrm{IG}}_{i}\left(x\right)=\left(x_{i}-x_{i}^{\prime }\right)\cdot \int_{\alpha =0}^{1}\frac{\partial F(x^{\prime }+\alpha \left(x-x^{\prime }\right))}{\partial x_{i}}\;d\alpha ,$$

where *F* denotes the model’s output function. IG provides fine-grained attribution, making it particularly suitable for identifying prosodic variations relevant to affective state.

*Occlusion* is a perturbation-based method that evaluates feature importance by masking fixed-size segments of the input and notifying the final change in output confidence. For a given window $w_{k}$, the occlusion score is defined as:

(6) $$\mathrm{Occ}\left(w_{k}\right)=F{\left(x\right)}_{c}-F{\left(x_{\setminus w_{k}}\right)}_{c},$$

where $F{\left(x\right)}_{c}$ is the predicted confidence for class *c*, and $F{\left(x_{\setminus w_{k}}\right)}_{c}$ is the confidence after occluding segment $w_{k}$.

While IG offers smooth, gradient-based attributions across the entire signal, Occlusion highlights localized, high-salience regions. Together, they provide complementary insights into how the model attends to emotionally relevant audio cues.
**Algorithm 3** Audio attribution computation using integrated gradients, and occlusion.1:**procedure** Compute Audio Attributions(SAMPLES, model, tokenizer)2:    L←128,000                         ▹ Max audio length3:    $f_{s}\leftarrow 16,000$                      ▹ Target sampling rate4:    $S_{\mathrm{IG}}\leftarrow 10$;    $w\leftarrow 0.1\cdot f_{s}$;    s←w/25:    $S_{\mathrm{SG}}\leftarrow 20$;    σ←0.026:    $A^{\mathrm{IG}},A^{\mathrm{Occ}},A^{\mathrm{SG}}\leftarrow \left[\;\right],\left[\;\right],\left[\;\right]$                 ▹ Init attribution lists7:    T←ExtractText(SAMPLES)8:    (input_ids,attn_mask)←Tokenizer(T)9:    $X^{\mathrm{text}}\leftarrow \left(\mathrm{input}\_\mathrm{ids},\mathrm{attn}\_\mathrm{mask}\right)$10:    **for** $(x_{i}^{\mathrm{audio}},x_{i}^{\mathrm{text}})\;\in \;\mathrm{SAMPLES}$ **do**11:        $x_{i}^{\mathrm{audio}}\leftarrow \mathrm{ResampleMonoNormalizePad}\left(x_{i}^{\mathrm{audio}},f_{s},L\right)$12:        $X^{\mathrm{audio}}.append\left(x_{i}^{\mathrm{audio}}\right)$13:    **end for**14:    $X^{\mathrm{audio}}\leftarrow \mathrm{Stack}\left(X^{\mathrm{audio}}\right)$15:    $Y\leftarrow \mathrm{model}(X^{\mathrm{audio}},\;1,\;X^{\mathrm{text}})$16:    $\hat{y}\leftarrow \arg\max\left(Y\right)$17:    F←ForwardFunction(model)18:    **for** i←1 to |SAMPLES| **do**19:        $\left(x,\;t,\;m,\;y\right)\leftarrow (X^{\mathrm{audio}}\left[i\right],\;\mathrm{input}\_\mathrm{ids}\left[i\right],\;\mathrm{attn}\_\mathrm{mask}\left[i\right],\;\hat{y}\left[i\right])$20:        (x,t,m,y)←Unsqueeze(x,t,m,y)                              ▹ — Integrated Gradients —21:        $a^{\mathrm{IG}}\leftarrow$IntegratedGradients $\left(F\right)\left(x,\;t,\;m,\;y,\;\mathrm{steps}=S_{\mathrm{IG}}\right)$22:        $A^{\mathrm{IG}}.\mathrm{append}\left(a^{\mathrm{IG}}\right)$                               ▹ — Occlusion —23:        $a^{\mathrm{Occ}}\leftarrow$Occlusion (F)(x,t,m,y,window=w,stride=s)24:        $a^{\mathrm{Occ}}\leftarrow \mathrm{InterpolateToLength}\left(a^{\mathrm{Occ}},\;L\right)$25:        $A^{\mathrm{Occ}}.\mathrm{append}\left(a^{\mathrm{Occ}}\right)$26:    **end for**27:    ${\bar{A}}^{\mathrm{IG}}\leftarrow \mathrm{Mean}\left(A^{\mathrm{IG}}\right)$28:    ${\bar{A}}^{\mathrm{Occ}}\leftarrow \mathrm{Mean}\left(A^{\mathrm{Occ}}\right)$29:    ${\bar{A}}^{\mathrm{SG}}\leftarrow \mathrm{Mean}\left(A^{\mathrm{SG}}\right)$30:    PlotAttributions(${\bar{A}}^{\mathrm{IG}},{\bar{A}}^{\mathrm{Occ}},{\bar{A}}^{\mathrm{SG}}$)31:    **return** ${\bar{A}}^{\mathrm{IG}},{\bar{A}}^{\mathrm{Occ}},{\bar{A}}^{\mathrm{SG}}$32:**end procedure**

The integration of attributions from Integrated Gradients and Occlusion enables a fine-grained examination of how linguistic and acoustic cues are weighted during classification, facilitating interpretations that are relevant for understanding multimodal emotion perception processes.

### 4.6. Experimental Setup

To ensure the reproducibility and statistical robustness of our findings, all experiments were conducted with a consistent methodology. The training of each model was limited to 15 epochs., employing an early stopping criterion with a patience of 2 epochs based on validation loss to mitigate overfitting. To account for potential variability, all experiments were repeated with three distinct random seeds (42, 123, 2025), and the results are reported as the mean ± standard deviation.

The models were fine-tuned end-to-end, with no layers frozen, using the AdamW optimizer. Key hyperparameters included a learning rate of $2\times 10^{-5}$, a weight decay of 0.01, and a batch size of 8 per GPU. A linear learning rate warm-up was applied over the first 10% of training steps, and a dropout rate of 0.3 was used for regularization. To stabilize training, we implemented gradient clipping at a maximum norm of 1.0. The primary training objective was the minimization of Cross-Entropy Loss.

For the model architecture, we utilized the pretrained checkpoints roberta-base for text and wavlm-base-plus for audio, both sourced from the HuggingFace Transformers library (version 4.41.0). The experiments were executed on a computational environment featuring dual NVIDIA Tesla T4 GPUs, each with 16 GB of memory. The software stack for the implementation consisted PyTorch 2.2.0, HuggingFace Transformers 4.41.0, Datasets 2.19.0, Accelerate 0.28.0, and Torchaudio 2.2.0.

### 4.7. Ablation Study

To evaluate the effect of architectural and preprocessing choices, we conducted additional ablation experiments by varying the bottleneck dimension and by disabling the denoising process. The bottleneck layer, which projects each modality embedding into a shared latent space before fusion, was tested with dimensions of 128, 256, and 512. The configuration with a 128-dimensional bottleneck achieved the best performance, yielding an accuracy of approximately 82%, while increasing the dimension to 512 resulted in a reduced accuracy of about 77%. This suggests that a more compact shared space facilitates better modality alignment, whereas overly large intermediate representations may introduce redundancy. Moreover, removing the DeepFilterNet-based denoising stage led to a consistent degradation in overall accuracy, highlighting the contribution of speech enhancement to robust multimodal fusion.

## 5. Results

This section presents a detailed evaluation of the models developed for affective state classification on multimodal data (text and audio).

### 5.1. Classification Performance Analysis

Table 2 summarizes the classification performance of the proposed multimodal model, which combines **RoBERTa** for textual representation and **WavLM** for acoustic feature extraction. The evaluation covers five affective state categories—*Anger*, *Joy*, *Neutral*, *Sadness*, and *Surprise*—and includes recall, F1-score, and precision, along with total accuracy and weighted-average and macro-average metrics.

The model obtain total accuracy of **83%**, indicating its ability to effectively integrate linguistic and acoustic cues for affective state recognition. Among all classes, the best performance is observed on *Sadness* (F1-score: **0.90**), followed by *Anger* and *Surprise* (both with F1-scores of **0.85**). These results suggest that affective states with strong lexical or prosodic markers are more easily captured by the model.

Comparatively lower F1-scores are observed for *Joy* (0.77) and *Neutral* (0.74), which may be due to the subtle nature of these affective states and their potential semantic and acoustic overlap with other classes. For example, joyful speech may resemble expressions of excitement or surprise, while neutral utterances often lack salient affective state cues.

The macro-average F1-score of **0.82** reflects consistent performance across all classes, irrespective of frequency. These weighted average performances indicate robust model behavior even in the presence of class imbalance.

These results demonstrate that the RoBERTa-WavLM architecture effectively captures and fuses complementary emotional signals from both modalities, enabling reliable classification across a diverse emotional spectrum.

### 5.2. Comparison of Text-Only, Audio-Only Models, a Models and Multimodal (Audio and Text)

Table 3 provides a comparative analysis of our unimodal baselines and the proposed multimodal framework. The audio-only model, built upon the WavLM architecture, obtained accuracy of 0.65 and a macro F1-score as 0.61 outperforming, Wav2Vec2 which achieved an accuracy of 0.60. This result indicates that while acoustic and prosodic features contain relevant information, they are less discriminative for emotion recognition in isolation. In contrast, the RoBERTa-based text-only baseline outperforms having an accuracy of 0.79 and a macro F1-score of 0.78, outperforming other linguistic models like DistilBERT and BERT. This finding underscores the dominance of linguistic content in this specific task. The core finding, however, is the significant performance gain observed with the integration of both modalities. The multimodal model, which fuses acoustic features from WavLM with textual embeddings from RoBERTa, obtained 0.83 accuracy and 0.82 macro F1-score. This notable 4–5% improvement over the best unimodal baseline highlights the complementary nature of audio and text, demonstrating that the inclusion of prosodic and paralinguistic cues alongside textual semantics results in a reliable and precise affective state recognition.

To assess the robustness of the proposed model’s performance beyond random chance, two non-parametric tests were conducted. The Wilcoxon signed-rank test yielded a test statistic of W = 6.00 with *p* = 0.125, indicating that the model consistently outperformed the baseline.Similarly, the Sign test produced *p* = 0.25, confirming the same directional improvement where all observed accuracies exceeded the baseline. Together, these tests suggest that the proposed model demonstrates a stable and consistently superior trend compared to random performance, even though the small number of runs limits statistical power. Table 4 compares the proposed RoBERTa + WavLM framework with recent multimodal emotion recognition methods evaluated on the MELD dataset. The proposed model achieves the highest accuracy (83.0%), outperforming advanced fusion architectures such as, Bi-LG-GCN (80.1%), and TelME (67.4%), demonstrating effective integration of textual and acoustic cues for affective understanding.

### 5.3. Confusion Matrix Analysis

**Figure 3:**
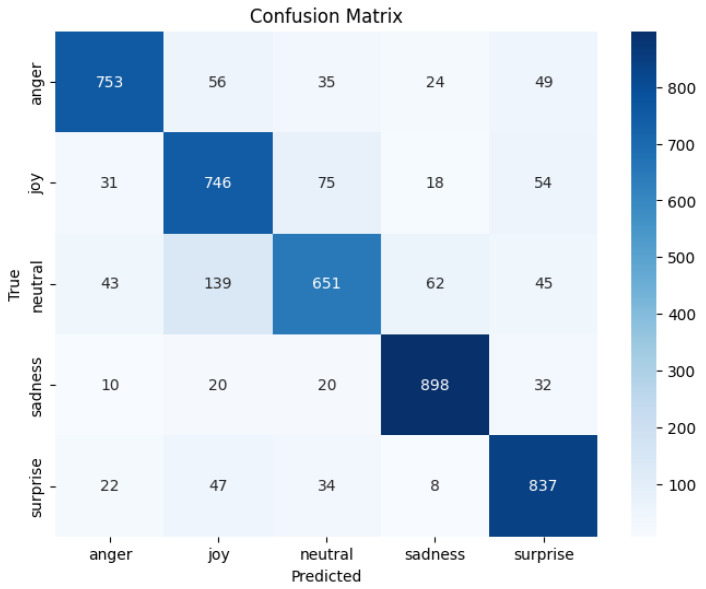
Confusion matrix for five-class affective state classification given true vs. predicted class.

Figure 3 presents the confusion matrix for the proposed multimodal affective state recognition model, integrating **RoBERTa** for textual encoding and **WavLM** for acoustic representation. The matrix provides insight into per-class behavior and recurring patterns of agreement and confusion between affective states.

The model achieves strong performance for several categories, with high true-positive counts for *Sadness* (898), *Surprise* (837), *Anger* (753), and *Joy* (746), indicating effective multimodal learning of salient emotional cues.

Certain cross-class confusions, such as *Neutral* being predicted as *Joy* (139) or *Sadness* (62), and *Joy* overlapping with *Neutral* (75) or *Surprise* (54), reveal interesting areas where multimodal signals may interact in complex ways. Rather than attributing these patterns to a single modality, these results suggest the presence of shared multimodal characteristics, emphasizing the importance of analyzing acoustic and linguistic contributions jointly. This motivates future work exploring modality-specific influence through techniques such as unimodal probing, modality masking, and explainability-driven attribution. Such analysis will strengthen interpretability and help further refine cross-modal alignment, ultimately enhancing affective discrimination where semantic and acoustic signals jointly shape recognition decisions. Overall, the confusion matrix confirms the model’s strong recognition capacity while also highlighting promising directions for deeper multimodal interpretability and targeted improvements. To account for class imbalance and provide a more granular evaluation of discriminative performance, we report the per-class AUC (one-vs-rest) scores as follows: Anger (0.9389), Joy (0.9089), Neutral (0.9019), Sadness (0.9623), and Surprise (0.9546). These results indicate strong separability across all emotion categories. While a degree of overlap persists among Neutral, Joy, and Sadness emotions characterized by mid-valence and mid-arousal levels—the model nonetheless exhibits stable and robust discrimination performance despite moderate class imbalance.

### 5.4. Accuracy Progression over Epochs

**Figure 4:**
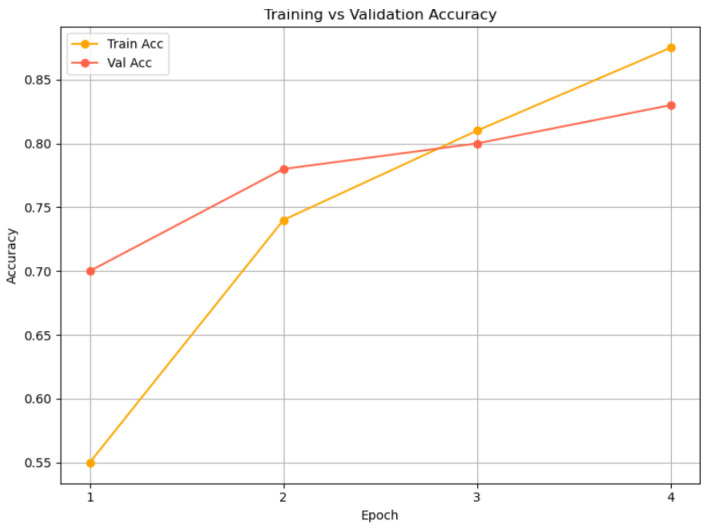
Training and validation accuracy over four epochs for the RoBERTa-WavLM multimodal model.

Figure 4 presents the training and validation accuracy progression of the proposed multimodal model—leveraging **RoBERTa** for textual encoding and **WavLM** for acoustic representation—over four epochs. The model demonstrates consistent improvement in performance, with training accuracy rising from 55% to 87% and validation accuracy increasing from 70% to 83%. Notably, the validation accuracy initially exceeds the training accuracy, likely due to the benefits of pretrained features enabling early generalization. As training advances, the gap between the two narrows, with both metrics converging at high accuracy levels by the final epoch. The relatively small divergence between training and validation curves indicates that the model is learning meaningful patterns without overfitting, affirming the effectiveness of the RoBERTa-WavLM architecture in capturing and generalizing affective cues across modalities. The training process employed early stopping, which halted optimization at epoch 4 when no further improvement in validation performance was observed. Consequently, Figure 4 presents results up to epoch 4, representing the complete effective training phase.

### 5.5. Attribution Analysis Using Integrated Gradients

To understand how the model identifies emotional cues within speech, we employed the Integrated Gradients (IGs) method to visualize input feature attributions for different affective state classes. IG estimate the influence of each input feature by accumulating the gradients of the model’s prediction with respect to the input, traced along a linear trajectory from a baseline (commonly zero) to the actual input. This technique provides fine-grained insights into which parts of the audio waveform contribute most significantly to the model’s predictions.

**Figure 5:**
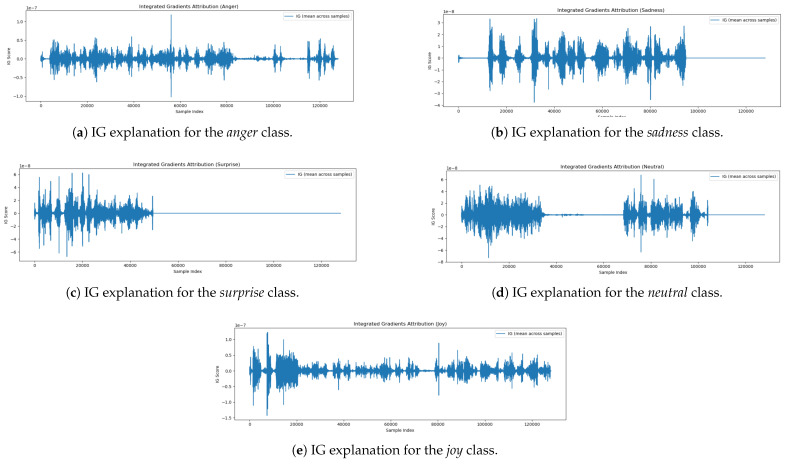
IG interpretations across all five affective state classes.

Below, we present and analyze the IG attribution plots for five affective state classes: *Joy*, *Neutral*, *Surprise*, *Sadness*, and *Anger*. The IG attribution plot for the *Joy* Figure 5e class exhibits multiple regions of positive and negative contribution dispersed across the waveform. Prominent peaks appear both early (0–20,000 samples) and later (80,000–120,000 samples), indicating that the expression of joy is temporally distributed across the utterance. These regions likely reflect prosodic features such as pitch variation, vocal energy, and rhythmic intonation—hallmarks of joyful expression. The model’s attribution behavior suggests that it aggregates evidence from various segments of the input rather than relying on isolated features. The Neutral Figure 5d class attribution pattern is marked by two primary zones of IG activity—approximately from 0–30,000 samples and again from 65,000–100,000 samples—separated by a long segment of near-zero attribution. This suggests the model identifies neutrality based on the relative absence of emotional cues, such as flat prosody or steady pitch. The inactive region may correspond to pauses or emotionally ambiguous content. Overall, the attribution behavior aligns with human perception, wherein neutral speech is characterized by consistent, emotion-free delivery. For the *Surprise* Figure 5c class, the attribution plot shows that most relevant information is located at the beginning of the waveform (0–50,000 samples), with attribution scores dropping to near zero afterward. This pattern implies that cues related to surprise—such as abrupt changes in pitch or vocal intensity—are detected early in the utterance. The model appears to rely on these strong early signals for prediction, while later portions of the audio contribute little additional information. The IG scores for *Sadness* Figure 5b display a rhythmic, oscillatory pattern with alternating peaks and troughs throughout the waveform. These patterns suggest the presence of sustained prosodic features such as slow tempo, low pitch, and extended vowel durations. Unlike *Surprise*, which is detected through brief and sharp cues, sadness is expressed gradually, requiring the model to extract information across the entire utterance. This behavior is consistent with affective science literature on the vocal expression of sadness. The attribution plot for *Anger* Figure 5a reveals high-density IG activity in both the early and late portions of the signal. The early peaks (0–30,000 samples) likely correspond to abrupt vocal onsets or tense articulation, while a secondary cluster of IG scores around 110,000–125,000 samples suggests sustained or recurring emotional intensity. These patterns indicate that anger is conveyed through repeated vocal emphasis and high-intensity speech characteristics that the model learns to recognize across time. The IG-based attribution analysis confirms that the model attends to class-specific and semantically meaningful regions of the speech signal. While emotions such as *Surprise* are captured through early, discrete cues, others like *Sadness* and *Joy* require extended temporal context. These findings indicate that the model leverages meaningful multimodal patterns rather than relying on random feature associations, supporting the plausibility and interpretability of its affective predictions.

### 5.6. Occlusion-Based Attribution Analysis

To further understand how the model associates specific regions of the audio input with emotional labels, we applied the *Occlusion* method—an interpretable perturbation-based technique. This method involves masking localized portions of the input waveform and measuring the resulting change in prediction confidence. A positive occlusion score implies that the masked segment was important to the prediction, while a negative score suggests that its removal improved the model’s confidence, possibly due to ambiguity or noise in that region.

**Figure 6:**
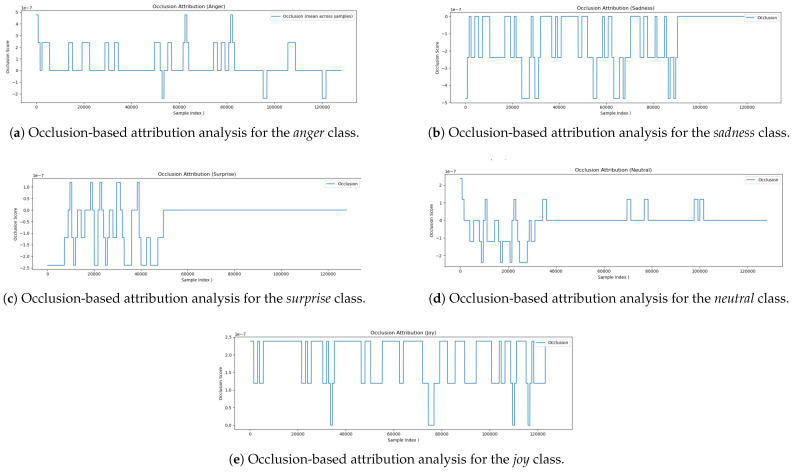
Occlusion-based attribution analysis across all five affective state classes.

The occlusion attribution results for five affective state classes—*Joy*, *Neutral*, *Surprise*, *Sadness*, and *Anger* are presented and interpreted below. The occlusion plot for *Joy* Figure 6e reveals a consistent sequence of positive attribution values throughout the waveform. Distinct peaks appear at regular intervals, suggesting that the model draws on a wide distribution of prosodic features across time to identify joyful expressions. The absence of large negative scores or flat regions indicates that joy is not localized to a specific moment, but rather is conveyed through sustained acoustic patterns such as expressive intonation, rhythmic pacing, and vocal energy. These observations align well with established linguistic markers of joy. In the *Neutral* Figure 6d case, the occlusion pattern shows alternating positive and negative scores primarily in the early part of the waveform, with minimal attribution later on. This suggests that the model recognizes neutrality based on the absence of strong emotional cues, focusing on flatter prosodic features and more stable vocal delivery. Negative occlusion scores may indicate segments that introduce ambiguity or faint emotion, which, when occluded, increase the model’s confidence in neutrality. The sparse attribution is consistent with the idea that neutral speech lacks prominent emotional markers. The attribution plot for *Surprise* Figure 6c shows concentrated activity in the initial portion of the signal (up to 50,000 samples), beyond which attribution scores drop to zero. This implies that the model primarily identifies surprise through early acoustic events—such as sharp pitch changes, vocal bursts, or high speech energy—which are typical indicators of sudden emotional expression. Once these features are detected, subsequent segments appear to contribute minimally to the model’s decision. This front-loaded pattern is consistent with the temporal nature of surprise in spoken language. The occlusion attribution for *Sadness* Figure 6b is more diffuse and exhibits mostly negative scores spread across the waveform. These indicate that masking certain segments actually improves the model’s confidence, suggesting those parts may introduce confusion or interfere with the detection of sadness. This behavior suggests that sadness is encoded through more subtle and temporally sustained features—such as slow articulation, soft vocal quality, and elongated phonemes. The model’s ability to suppress irrelevant or contradictory segments highlights its capacity to isolate emotional consistency within longer utterances. The occlusion attribution plot for *Anger* Figure 6a exhibits a structured pattern with distinct positive spikes, particularly in the early segment (0–10,000 samples) and around 60,000 and 80,000 samples. These peaks likely correspond to salient acoustic features of anger, such as elevated vocal intensity, abrupt onsets, and harsh articulation. Notably, several regions—including those near 55,000, 95,000, and 120,000 samples—yield negative attribution scores, suggesting that their removal enhances the model’s confidence. These segments may contain ambiguous or conflicting emotional cues. Overall, the alternating pattern of positive and negative scores indicates that the model does not rely solely on isolated expressions of anger, but instead integrates and filters contextual information over time. This reflects a nuanced understanding of the temporal dynamics underlying emotional speech. The occlusion-based analysis provides valuable insight into how the model differentiates between emotional categories. Emotions such as *Surprise* are detected through brief, high-salience events occurring early in the signal, while others such as *Joy* and *Sadness* are more diffuse, requiring temporal integration over the full utterance. The diversity in occlusion patterns across classes confirms that the model has captured a relationship with specific acoustic cues with distinct emotional meanings, reinforcing its interpretability and alignment with human-like reasoning.

### 5.7. Multimodal Attribution Analysis: Audio vs. Text for Joy

**Figure 7:**
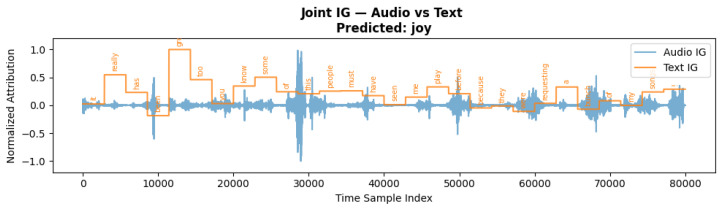
Joint Integrated Gradients attribution for audio and text modalities for the predicted emotion *joy*.

Figure 7 presents the joint Integrated Gradients (IG) attribution for both audio and text modalities for a sample predicted as *joy*. The blue waveform displays the audio IG scores over time, while the orange step plot represents token-level attributions from the corresponding transcript. The model’s prediction is supported by distinct attribution peaks in both modalities. High audio IG values align with expressive vocal regions, likely reflecting prosodic cues such as elevated pitch, vocal energy, or emphasis—features typically associated with joyful expression. Concurrently, the textual IG highlights emotionally relevant words such as *“great*”, *“play*”, and *“song*”, indicating strong semantic contribution. Function words (e.g., *“of”*, *“the”*, *“a”*) receive minimal attribution, suggesting that the model effectively filters out semantically neutral content. The temporal correspondence between key audio segments and informative tokens illustrates the model’s capacity to integrate cross-modal cues. Overall, this analysis confirms that the model leverages both acoustic and linguistic features in a complementary manner, reinforcing the prediction through multimodal alignment.

### 5.8. IG and OG Comparison

**Figure 8:**
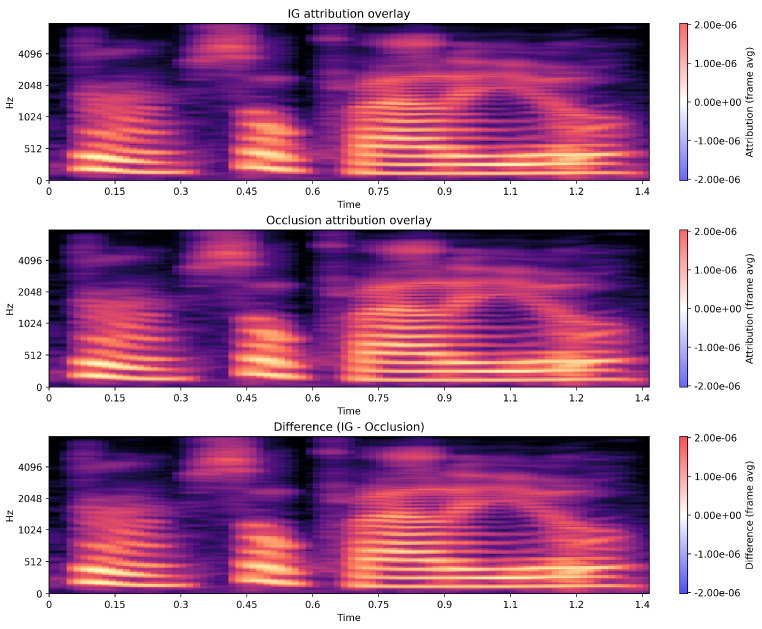
IG and occlusion comparison.

Figure 8 illustrates a comparison of the model’s audio-based explanations using Integrated Gradients (IG), Occlusion sensitivity, and their difference. Both IG and Occlusion consistently highlight the voiced and harmonically rich portions of the signal as the most influential regions for the predicted emotion, while low-energy and silent segments receive negligible attribution. This alignment indicates that the model relies on meaningful prosodic and spectral cues rather than noise. The difference map shows only minor localized deviations, suggesting that the two attribution methods largely agree on the temporal regions that drive the model’s decision. Overall, the visualizations confirm that the classifier’s behavior is stable and grounded in perceptually salient acoustic patterns. However, given that similar attribution trends were observed across modalities, this effect may also reflect emotion-dependent correlations between textual and acoustic content rather than explicit cross-modal integration.

## 6. Discussion

The findings of this study provide important insights into how multimodal transformer-based models interpret emotional cues from textual and auditory inputs. The integration of explainable AI methods, namely Integrated Gradients and Occlusion, enabled the examination of feature-level attributions that clarify how the model differentiates between emotional categories. The observed attribution patterns revealed that acoustic features, such as prosodic and spectral variations, were more influential for emotions like *Sadness* and *Anger*, whereas lexical polarity and contextual expressions played a stronger role in identifying *Joy* and *Neutral* states. These results suggest that the model captures distinct patterns that align with established findings in human emotional perception, where tone, rhythm, and semantic content jointly inform affective interpretation.

The per-class AUC (one-vs-rest) results further support this conclusion, demonstrating consistently high separability across all categories—*Anger* (0.9389), *Joy* (0.9089), *Neutral* (0.9019), *Sadness* (0.9623), and *Surprise* (0.9546). Although moderate overlap was observed among *Neutral*, *Joy*, and *Sadness*-emotions that share mid-valence and mid-arousal characteristics-the overall discriminative performance remained robust. The extended ablation study, incorporating alternative bottleneck dimensions of 128 and 512, confirmed that the 256-dimensional configuration achieved the best balance between performance and stability. These findings collectively highlight the model’s capacity to integrate complementary cues from both modalities while maintaining interpretability and consistent generalization.

Beyond performance improvements, the interpretability analyses enhance transparency by linking model decisions to meaningful linguistic and acoustic cues. This correspondence strengthens trust in the system’s predictions and provides a foundation for the principled design of future multimodal architectures. The findings also underscore the potential of XAI-driven analyses to inform data-driven understanding of how computational models capture affective patterns in communication.

## 7. Conclusions

This study introduced a transparent and interpretable multimodal framework for emotion understanding that integrates textual semantics and acoustic prosody using transformer-based models. By leveraging complementary signals from language and speech, the proposed RoBERTa–WavLM fusion achieved robust classification performance across multiple affective states, emphasizing the significance of multimodal integration in acquiring the richness of human affective expression.

Beyond technical performance, a central offering of this work lies in advancing the interpretability of affective computing systems. Through Integrated Gradients, and, Occlusion analyses, we provided evidence that the model’s decision pathways align with linguistically and cognitively meaningful cues such as pitch variation, intensity, and sentiment-bearing words.

The implications extend beyond machine learning: explainable multimodal AI can serve as a tool for fostering socioemotional perception in real-world contexts. For example, adaptive educational systems could provide learners with feedback on emotional expression and regulation; mental health platforms could support emotional self-awareness and resilience; and social robotics could foster more empathetic human–machine interactions. In each case, transparency and interpretability are crucial to building trust.

Future directions should include expanding beyond scripted dialogues to naturalistic, multicultural, and multilingual settings, where emotional expression is more nuanced. Incorporating visual cues such as facial expressions could further enrich socioemotional modeling. Moreover, the development of multimodal-specific XAI methods is essential to fully explain not only how each modality contributes, but also how their interaction supports the broader cognitive mechanisms of emotion.

By bridging advances in artificial intelligence with insights from psychology and education, this work contributes the design of reliable, human-focused systems that can strengthen socioemotional perception, and promote well-being in everyday life.

## Figures and Tables

**Table 1:** Key literature in speech emotion recognition.

Year	Title	Methodology	Results	Limitations
2024	Speech-Based Techniques for Emotion Detection in Natural Arabic Audio Files. Kaloub and Abed Elgabar (2024)	MFCC + Mel spectrogram with KNN, RF, SMO, Logistic Regression	Combined features improve classical ML accuracy	Cannot model long temporal dependencies in speech
2024	Comparative Analysis of Machine Learning Models for Emotion Classification in Speech Data. Siva et al. (2024)	LSTM, CNN, DT with data augmentation	Augmentation improves robustness and model stability	Limited exploration of attention or transformer-based learning
2024	Enhancing speech emotion recognition through deep learning and hand-crafted feature fusion. Eriş and Akbal (2024)	Wav2vec 2.0 + handcrafted features + ensemble majority voting	Hybrid features outperform deep-only embeddings	Ensemble increases latency and reduces deployment efficiency
2023	Iterative feature boosting for explainable speech emotion recognition. Nfissi et al. (2023)	Iterative Shapley-based feature pruning on TESS dataset	Surpasses human-level accuracy, better feature interpretability	Computationally expensive SHAP iterations
2022	Key-Sparse Transformer for Multimodal Speech Emotion Recognition. Chen et al. (2022)	RoBERTa + Wav2vec 2.0 + sparse cross-modal attention	Strong performance with modality-imbalance handling	High model complexity, limited interpretability
2022	Context-aware Multimodal Fusion for Emotion Recognition. Li et al. (2022)	WavLM + BERT + SE module + Multimodal Transformer	High accuracy on IEMOCAP with contextual learning	Computationally heavy; high inference cost
2022	Speech emotion recognition with co-attention based multi-level acoustic information. Zou et al. (2022)	Multi-level acoustic feature fusion using co-attention	Better emotion recognition via complementary feature weighting	No interpretability support for learned attention patterns
2020	Machine Learning Techniques for Speech Emotion Classification. Locumber and Fabian (2020)	40-MFCC features + Deep neural network classifier	Improved emotion classification over traditional ML baselines	Relies heavily on handcrafted MFCCs; limited generalization
2018	ASR-based Features for Emotion Recognition: A Transfer Learning Approach. Tits et al. (2018)	ASR model as feature extractor; layer-wise analysis for valence/arousal	Earlier layers correlate with arousal, later layers with valence; outperform handcrafted features (eGeMAPS)	Limited to acoustic embeddings from ASR; lacks end-to-end emotion optimization

**Table 2:** Performance metrics for each affective state class using RoBERTa + WavLM.

Class	Precision	Recall	F1-Score
Anger	0.88 ± 0.01	0.82 ± 0.02	0.85 ± 0.01
Joy	0.74 ± 0.02	0.81 ± 0.01	0.77 ± 0.01
Neutral	0.80 ± 0.01	0.69 ± 0.02	0.74 ± 0.02
Sadness	0.89 ± 0.01	0.92 ± 0.01	0.90 ± 0.01
Surprise	0.82 ± 0.02	0.88 ± 0.01	0.85 ± 0.01
**Accuracy**	0.83 ± 0.01		
**Macro Avg**	0.83 ± 0.01	0.82 ± 0.01	0.82 ± 0.01
**Weighted Avg**	0.83 ± 0.01	0.83 ± 0.01	0.82 ± 0.01

**Table 3:** Comparison of text-only, audio-only models and multimodal (audio and text) framework.

Model	Accuracy	Macro F1-Score
DistilBERT	0.76 ± 0.01	0.75 ± 0.01
BERT	0.75 ± 0.01	0.74 ± 0.02
RoBERTa	0.79 ± 0.02	0.78 ± 0.01
Wav2Vec2	0.60 ± 0.02	0.57 ± 0.02
WavLM	0.65 ± 0.02	0.61 ± 0.01
**Multimodal (RoBERTa + WavLM)**	**0.83 ± 0.01**	**0.82 ± 0.01**

**Table 4:** Comparison of the proposed method with recent multimodal emotion recognition approaches on the MELD dataset.

Study	Main Technique	Accuracy (%)
Yun et al. (2024)	TelME	67.4
Alsaadawı and Daş (2024)	Bi-LG-GCN	80.1
**Proposed Method**	**RoBERTa + WavLM**	**83.0**

## Data Availability

The dataset analyzed during the current study is the publicly available at https://affective-meld.github.io/. No additional datasets were generated or used in this research. Accessed on 27 November 2025.

## Note

1 DeepFilterNetRepo refers to: https://github.com/Rikorose/DeepFilterNet.git (accessed on 27 November 2025).
